# Innovative microneedle-integrated hydrogels: a promising strategy for diabetic foot ulcer management

**DOI:** 10.3389/fbioe.2026.1826873

**Published:** 2026-07-01

**Authors:** Haiyang Yang, Haodong Hu, Yuhao Wang, Yiquan Ji, Jun Ma, Zhuoming Xu, Kang Ji, Jiayi Chen, Huanhuan Luo, Gang Chen

**Affiliations:** 1 Jiaxing University Master Degree Cultivation Base, Zhejiang Chinese Medical University, Hangzhou, China; 2 Department of Orthopedics, Jiaxing Key Laboratory of Basic Research and Clinical Translation on Orthopedic Biomaterials, The Second Affiliated Hospital of Jiaxing University, Jiaxing, China

**Keywords:** diabetic foot ulcers, microneedle (MN), microneedle-based hydrogel, targeted drug delivery, wound repair and regeneration

## Abstract

Diabetic foot ulcer (DFU) is a common and severe complication in diabetic patients, characterized by prolonged wound healing, susceptibility to infection, and a high risk of amputation. Its complex pathophysiology involves persistent hyperglycemia, chronic inflammation, neuropathy, vascular impairment, infection, and cellular dysfunction. Conventional treatments often fall short in addressing these multifaceted challenges, Therefore, formulating safe and effective treatment strategies holds significant clinical significance. In recent years, Microneedle Hydrogels have emerged as a novel drug delivery system due to their minimally invasive application, high drug loading capacity and controlled release characteristics, This review highlights the unique advantages of Microneedle Hydrogel systems, including their structural characteristics, drug delivery capabilities, and mechanisms in promoting ulcer healing. At the same time, the challenges currently faced in research and the future development directions were explored, aiming to provide references for further research and clinical application in this field.

## 1 Introduction

Diabetic foot ulcers (DFU) represent a severe and prevalent global complication of diabetes mellitus, afflicting an estimated 33 million individuals worldwide, corresponding to a prevalence of approximately 6.3% among people with diabetes (Mcdermott et al., 2023; Zhang et al., 2017), represent a substantial global burden, imposing substantial morbidity including pain and delayed healing. They incur significant healthcare expenditures and considerable economic burden (Mcdermott et al., 2023). The recalcitrant nature of DFUs frequently leads to complications such as infection and gangrene, which may ultimately necessitate limb amputation. This progression results in devastating consequences for patients, including loss of functional independence, diminished quality of life, and adverse psychological sequelae. Conventional treatments such as debridement, antibiotic therapy, (Kumar et al., 2026),and wound dressing changes often yield limited results (Li et al., 2023; Liu et al., 2022; Cai et al., 2024), highlighting the urgent need for novel therapeutic strategies to improve patient outcomes. As an emerging treatment approach, microneedle hydrogels combine the penetrating ability of microneedles with the drug-delivery properties of hydrogels, effectively overcoming the stratum corneum barrier to enable precise and sustained drug release (Zhong et al., 2026; Wang et al., 2026).

Recent studies have shown that microneedle hydrogels hold great potential in the treatment of diabetic foot. For instance, one study indicated that microneedle hydrogels can significantly increase local drug concentration through sustained drug release, thereby promoting wound healing and reducing infection rates (Abid Al-Wahaab and Al-Mayahy, 2024). Additionally, the biocompatibility and tunability of microneedle hydrogels make them particularly promising for diabetic foot therapy. It is worth noting, however, that the preparation and performance optimization of microneedle hydrogels remain key challenges in current research.

Although significant progress has been made in the application of microneedle hydrogels for diabetic foot treatment, several challenges remain, including material optimization, scalable production, and clinical translation. Studies indicate that material selection and microneedle design play a decisive role in drug release efficiency and therapeutic outcomes. Furthermore, the clinical application of microneedle hydrogels must also consider patient acceptance and ease of use to ensure their effectiveness and feasibility in real-world treatment scenarios.

Over the past decade, research interest in microneedles for wound healing has grown exponentially. However, studies focusing on diabetic wounds remain relatively limited, indicating significant potential for further development in this field. This review aims to provide a reference for future research by highlighting recent advances in microneedle-based approaches for diabetic wound healing. While some reviews have discussed research and developments in diabetic wound healing, these works primarily focus on broad microneedle technologies or general transdermal applications. In contrast, this review specifically emphasizes studies on the mechanisms of diabetic wound healing using developed microneedle systems, integrating the latest progress from the past 5 years. By linking preparation methods and material design with the pathological mechanisms unique to diabetic wounds, this review offers an updated and more targeted perspective, complementing and expanding the scope of earlier publications. We introduce the pathogenesis of diabetic wounds, the wound healing process, and the main challenges currently faced, along with the application of microneedles in treatment. Finally, we discuss the current challenges and future research directions for the clinical translation of these microneedle technologies.

## 2 Pathophysiological characteristics and therapeutic challenges of diabetic foot

### 2.1 Wound healing process

**FIGURE 1:**
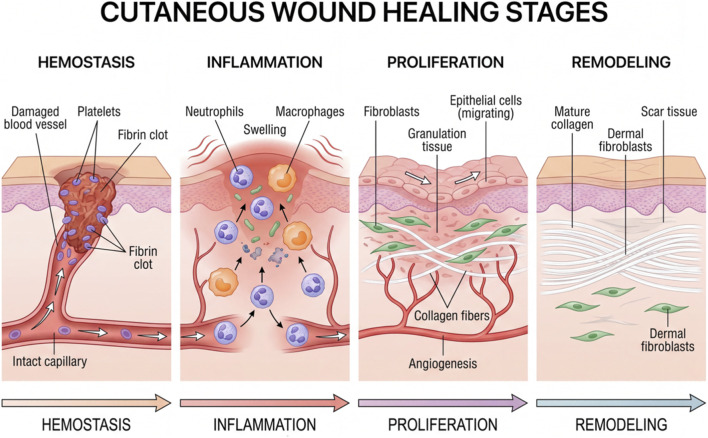
Overview of Wound healing.

Wound healing is a highly complex and coordinated biological process aimed at restoring the integrity and function of damaged tissues. This process is typically divided into four continuous and overlapping stages: hemostasis, inflammation, proliferation, and remodeling (Ebner and Maytin, 2009; Mamun et al., 2024; Akhtari et al., 2024). These stages are driven by the precise interactions of various cell types, cytokines, growth factors, and extracellular matrix components (Mamun et al., 2024; Akhtari et al., 2024) (Figure 1).

#### 2.1.1 Hemostatic phase

The initial stage of wound healing is hemostasis, which begins immediately after injury occurs. Vasoconstriction reduces blood loss, followed by the activation and aggregation of platelets at the injury site to form a platelet plug (Akhtari et al., 2024; Sharma Y. et al., 2024). Platelets release various bioactive molecules, such as growth factors and cytokines, which not only contribute to hemostasis but also initiate the subsequent inflammatory response, laying the foundation for the following stages (Mamun et al., 2024; Akhtari et al., 2024).

#### 2.1.2 Inflammatory stage

The inflammatory phase lasts approximately 1–3 days and is characterized by the release of serotonin and histamine from mast cells, which increases local vascular permeability at the wound site. This promotes the migration of neutrophils, monocytes, and chemokines to the injured area, initiating the inflammatory response (Akhtari et al., 2024). Neutrophils are the first immune cells to arrive at the wound site, where they phagocytose bacteria and cellular debris, and release proteases and reactive oxygen species (ROS) to clear damaged tissue (Criollo-Mendoza et al., 2023; Liu et al., 2025). Around 24–48 h after injury, monocytes are recruited to the wound site and differentiate into macrophages (Danella et al., 2023). These macrophages primarily exhibit the classically activated M1 phenotype, producing large amounts of pro-inflammatory cytokines such as tumor necrosis factor-alpha (TNF-α) and interleukin-1β (IL-1β) to enhance bactericidal and phagocytic functions, thereby sustaining the inflammatory state (Pereira Beserra et al., 2020). Subsequently, macrophages engulf a significant number of apoptotic neutrophils, a key signal that drives their transition toward the pro-repair M2 phenotype (Liu et al., 2025). M2 macrophages secrete anti-inflammatory cytokines, such as interleukin-10 (IL-10), as well as various growth factors that drive repair during the proliferative phase, including transforming growth factor-beta (TGF-β) and vascular endothelial growth factor (VEGF) (Pereira Beserra et al., 2020; King et al., 2014). This shift from M1 to M2 serves as a critical regulatory switch linking the inflammatory response to the tissue proliferative phase, suppressing inflammation and initiating the next stage—the proliferative phase (Mamun et al., 2024; Liu et al., 2025).

#### 2.1.3 Proliferation phase

The proliferative phase generally spans from day 4 to day 21. During this period, lymphocytes and macrophages migrate to the wound site to assist in controlling infection and removing cellular debris (Lacina et al., 2024; Landén et al., 2016). This stage is crucial for tissue repair and is marked by the development of granulation tissue, re-epithelialization, and angiogenesis (Akhtari et al., 2024; Ma et al., 2022). Under the influence of growth factors such as TGF-β secreted by M2 macrophages, fibroblasts migrate into the wound area and undergo extensive proliferation (Chen et al.). These cells act as the primary producers of the extracellular matrix (ECM), synthesizing and secreting substantial amounts of new ECM components—primarily type III collagen (Liu et al., 2025; Landén et al., 2016), fibronectin, and glycosaminoglycans—which form the structural framework of the granulation tissue. Granulation tissue, characterized by an abundance of new blood vessels, fibroblasts, and a loose extracellular matrix, serves as an indicator of effective tissue repair. Under persistent mechanical tension and TGF-β signaling, a subset of fibroblasts differentiates into myofibroblasts (Landén et al., 2016; Chen et al., 2026). These specialized cells exhibit hybrid characteristics of fibroblasts and smooth muscle cells and play a key role in wound contraction. A sufficient blood supply is vital to sustain cellular proliferation and matrix synthesis within the granulation tissue. Angiogenesis is triggered by hypoxic conditions at the wound center, where hypoxia stabilizes hypoxia-inducible factor-1α (HIF-1α) (Liu et al., 2025; Landén et al., 2016), leading to the upregulation of vascular endothelial growth factors growth factor-A (VEGF-A) expression ession (Liu et al., 2025). VEGF-A establishes a chemical gradient that guides endothelial cells from adjacent blood vessels to grow toward the wound center, forming new capillaries. Once the nascent vessels stabilize, endothelial cells secrete PDGF-B (Liu et al., 2025), recruiting pericytes to envelop the vessels and reinforce their structure. When the tissue defect is filled with granulation tissue, the healing process enters the prolonged remodeling phase (Mamun et al., 2024; Landén et al., 2016).

#### 2.1.4 Reshaping phase

The remodeling phase generally commences around 21 days post-injury and is characterized by the dynamic restructuring of the extracellular matrix (ECM) (Landén et al., 2016). In this stage, the initially deposited fine and disorganized type III collagen within the granulation tissue is progressively degraded by matrix metalloproteinases (MMPs) (Akhtari et al., 2024). It is subsequently replaced by thicker, more robust type I collagen produced by fibroblasts. The proteolytic activity of MMPs is precisely modulated by their endogenous inhibitors—tissue inhibitors of metalloproteinases (TIMPs) (Akhtari et al., 2024; Chen et al.). The equilibrium between MMPs and TIMPs governs whether net synthesis or degradation of the ECM occurs (Akhtari et al., 2024; Landén et al., 2016). As wound maturation proceeds, profibrotic signaling subsides, mechanical tension declines (Kamal et al., 2024), and a significant proportion of myofibroblasts undergo apoptosis. Simultaneously, neovascularization recedes, resulting in scar tissue that gradually becomes pale and sparsely populated by cells. The ultimate outcome is the formation of mature scar tissue (Mamun et al., 2024).

### 2.2 The healing process of diabetic wounds

Compared to normal wound healing, the hemostatic function in diabetic foot ulcers (DFU) may be impaired due to vascular pathology and platelet dysfunction (Lu et al., 2025; Cai et al., 2023). Although the initial hemostatic process still occurs, diabetes-induced vascular endothelial dysfunction and coagulation abnormalities can affect the stability and quality of thrombus formation, thereby indirectly influencing the initiation of subsequent inflammatory responses (Lu et al., 2025).

The inflammatory phase in diabetic foot ulcers is characterized by a persistent inflammatory reaction. Hyperglycemia leads to impaired immune cell function, reducing the chemotactic, phagocytic, and bactericidal capabilities of neutrophils and macrophages. Local levels of pro-inflammatory cytokines, such as tumor necrosis factor-alpha (TNF-α) and interleukin-1β (IL-1β), remain elevated, while anti-inflammatory factors like interleukin-10 (IL-10) are relatively insufficient, resulting in excessive and prolonged inflammation (Cai et al., 2023). Impaired transition from M1 to M2 macrophages further hinders effective clearance of pathogens and necrotic tissue (Lu et al., 2025; Cai et al., 2023).

In a hyperglycemic environment, fibroblast proliferation and migration are suppressed (Heisler et al., 2024; Roy and Gorain, 2025), and the synthesis of collagen and other extracellular matrix components is reduced (Heisler et al., 2024), leading to poor granulation tissue quality and an inability to adequately fill tissue defects. Impaired differentiation and contractile function of myofibroblasts also contribute to delayed wound healing (Heisler et al., 2024).

Elevated tissue glucose levels cause vascular pathology, endothelial dysfunction, and insufficient expression of local growth factors such as vascular endothelial growth factor (VEGF), severely impairing angiogenesis. Newly formed blood vessels are fragile and dysfunctional, failing to supply adequate oxygen and nutrients to the wound, which further exacerbates impaired healing (Hosseinzadeh et al., 2023).

The JAK/STAT signaling pathway is activated in chronic wound healing and regulates cell proliferation, differentiation, and immune responses (Kamal et al., 2024; Jere et al., 2017). However, in diabetic wounds, abnormal activation of these pathways may further disrupt the healing process. Additionally, an imbalance between collagen synthesis and degradation, along with a dysregulated ratio of matrix metalloproteinases (MMPs) to tissue inhibitors of metalloproteinases (TIMPs), leads to excessive MMP activity and subsequent over-degradation of the extracellular matrix, ultimately resulting in prolonged wound healing (Kamal et al., 2024).

### 2.3 Pathogenesis of diabetic foot ulcer

**FIGURE 2:**
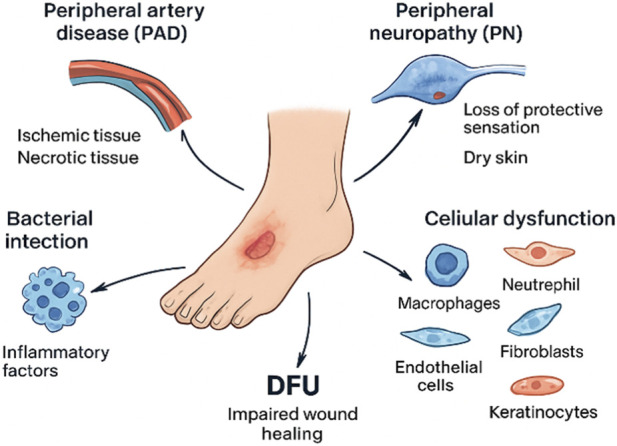
The four main aspects of diabetic foot ulcer formation: peripheral arterial disease, peripheral neuropathy, bacterial infection, and cell dysfunction.

Diabetic foot is a severe complication of diabetes mellitus characterized by a multifactorial pathophysiology. Key mechanisms include chronic hyperglycemia-induced microvascular dysfunction, peripheral neuropathy, and a dysregulated wound microenvironment (Mcdermott et al., 2023; Armstrong et al., 2017; Villa et al., 2024; Davis et al., 2018).Additional contributing factors involve impaired immune responses and the formation of bacterial biofilms, which further complicate wound healing and infection control (Mcdermott et al., 2023; Deng et al., 2023). The key pathogenic factors involved in the development of diabetic foot are illustrated in Figure 2.

#### 2.3.1 Impact of chronic hyperglycemia on the healing of diabetic foot ulcers

Chronic hyperglycemia represents a key etiological factor in the development of diabetic foot. Mechanistically, elevated blood glucose concentrations facilitate the formation of advanced glycation end products (AGEs), which subsequently accumulate in various tissues and induce structural and functional alterations in proteins, resulting in increased stiffness of the vasculature (Huijberts et al., 2008; Goldin et al., 2006; Forbes and Cooper, 2013). Numerous high-quality studies demonstrate that advanced glycation end products (AGEs) can induce endothelial dysfunction, accelerate atherosclerosis, and promote microvascular thrombosis, collectively increasing the susceptibility to diabetic foot (Elattar et al., 2023; Choonara, 2024). Concurrently, diabetes-associated neuropathy—primarily distal symmetric polyneuropathy—diminishes protective sensation, rendering patients unable to perceive minor traumatic injuries. Consequently, unrecognized wounds frequently progress to chronic ulcerations, establishing a pathway for diabetic foot complication (Lv et al., 2024; Liu Y. T. et al., 2023).

#### 2.3.2 Impact of the immune microenvironment on diabetic foot ulcer healing

The diabetic foot wound microenvironment plays a critical role in impairing the healing process (Villa et al., 2024). Chronic inflammation can trigger extensive infiltration of inflammatory cells, dysregulated overproduction of pro-inflammatory cytokines, and markedly elevated synthesis of proteolytic enzymes. Collectively, these pathological processes contribute to extracellular matrix degradation, functional impairment of growth factors, and substantial suppression of cellular proliferation, angiogenesis, and tissue regeneration (Chen et al., 2022). These pathological states are characterized by persistent inflammatory activation, abnormal angiogenesis and dysregulated extracellular matrix remodelling; these processes act in concert to maintain a persistent inflammatory state (Forbes and Cooper, 2013; Chang and Nguyen, 2021; Qi et al., 2023). This chronic inflammation disrupts the normal healing cascade, leading to significantly delayed wound closure and increased susceptibility to secondary infections (Elattar et al., 2023; Hafeez et al., 2022). Notably, studies have demonstrated a direct correlation between prolonged infiltration of pro-inflammatory cells, dysregulated cytokine secretion, and impaired tissue regeneration in diabetic foot ulcers (Chaitra et al., 2025).

#### 2.3.3 Impact of bacterial infection on the healing of diabetic foot ulcers

The formation of bacterial biofilms represents another critical characteristic of diabetic foot infections (Villa et al., 2024; He et al., 2023). Biofilms develop as microbial communities encased within a protective extracellular matrix, which adheres to wound surfaces (Pouget et al., 2020). This structure significantly enhances antibiotic resistance and fosters the emergence of multidrug-resistant strains (Ji et al., 2022; Stewart and William, 2001; Yin et al., 2019). In diabetic foot ulcers, bacterial biofilms not only shield pathogens from the host immune system but also impede wound healing and elevate the risk of persistent infection. In severe cases, these infections can lead to amputation (Elattar et al., 2023; Liu Y. T. et al., 2023; Cavanagh et al., 2005).

In summary, the pathophysiological mechanisms of diabetic foot are multifaceted, involving microvascular and nerve damage caused by chronic hyperglycemia, abnormalities in the wound microenvironment, and the formation of bacterial biofilms, among others. Research targeting these pathological mechanisms will provide more effective strategies for the prevention and treatment of diabetic foot.

### 2.4 Limitations of current treatment methods

**FIGURE 3:**
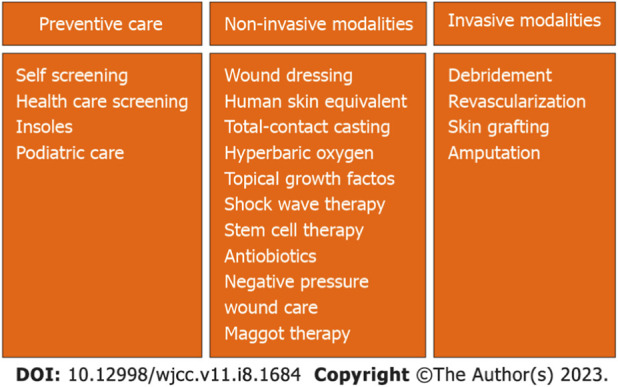
Treatment methods for diabetic foot ulcers.

Impaired wound healing among individuals with diabetes contributes to substantial morbidity and mortality, with potentially significant socioeconomic consequences.The prevention of complications and efficient healing of diabetic foot ulcers are contingent on regular monitoring, patient education, and multidisciplinary care. In severe cases, surgical intervention, such as amputation, may be necessary. These techniques are classified in Figure 3 as non-invasive and invasive modalities for a clear overview (Raja et al., 2023). Although various treatment options exist for diabetic foot ulcers, current therapeutic strategies are substantially limited by suboptimal efficacy and procedural complexity.

First, conventional wound dressings, such as gauze and foam-based materials, primarily serve as physical barriers but exhibit inadequate permeability, which hinders the effective delivery of macromolecular therapeutics. This limitation is particularly problematic in treatments requiring localized high drug concentrations, thereby restricting their overall therapeutic potential (Wang et al., 2022; Gao et al., 2022).

Secondly, systemic drug delivery often results in low local bioavailability, failing to achieve therapeutically effective drug concentrations at the wound site (Everett and Mathioudakis, 2018). This is further exacerbated by the metabolic disturbances in diabetic patients, which impair drug absorption and distribution (Drucker, 2020; Peng et al., 2023). Conventional routes of administration, such as oral or intravenous delivery, frequently fail to maintain adequate drug levels in the affected tissue, thereby prolonging the healing process (Choonara, 2024; Lashkarbolouk et al., 2023; Sung et al., 2012). Consequently, systemic administration alone is insufficient to address the complex therapeutic challenges of diabetic foot ulcers.

Furthermore, treatment challenges include secondary injury resulting from frequent dressing changes and suboptimal patient compliance (Ghanassia et al., 2008). On the one hand, frequent changes to wound dressings not only exacerbate the patient’s discomfort but also increase the risk of mechanical injury and infection at the wound site (Jiang et al., 2023; Mariadoss et al., 2022). On the other hand, the need for regular clinic visits and frequent dressing changes often leads to reduced treatment adherence due to pain and practical inconvenience, thereby adversely affecting the overall treatment outcome.

In summary, current therapeutic approaches for diabetic foot ulcers remain substantially limited by inefficient drug delivery, suboptimal systemic administration, and poor patient adherence (Jia et al., 2025; Omotosho et al., 2025). Future research should prioritize the development of advanced drug delivery systems—such as microneedle-integrated hydrogels—to enhance local drug concentration and bioavailability, thereby improving treatment outcomes (Fuentevilla-Alvarez et al., 2024; Dwivedi et al., 2024). It is anticipated that such innovative strategies will contribute to more effective and safer clinical management of diabetic foot ulcers.

## 3 Technical characteristics of hydrogel microneedles and their advantages for diabetic foot treatment

### 3.1 Functional characteristics and advantages of microneedle hydrogels

**FIGURE 4:**
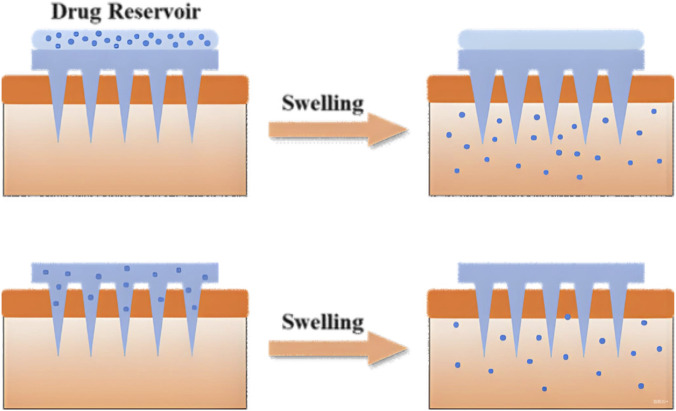
Schematic diagram of hydrogel microneedle drug delivery.

Hydrogel microneedle technology is an innovative drug delivery strategy that combines the advantages of transdermal microneedles with the functional properties of hydrogel materials (Ahmed Saeed Al-Japairai et al., 2020; Zhou et al., 2025). Microneedles are typically designed with micrometer-scale needle structures, allowing them to effectively breach the stratum corneum without stimulating underlying nerve endings, thereby markedly reducing patient discomfort during administration (Pratap and Singh, 2025; Liu et al., 2024a). Furthermore, the high-density array configuration of microneedles greatly increases the surface area available for drug delivery, enhancing overall drug administration efficiency (Zhang R. et al., 2025). The high biocompatibility, tunable degradation characteristics, and excellent drug-loading capacity of hydrogel materials facilitate controlled drug release and adaptability to diverse therapeutic scenarios (Ahmed Saeed Al-Japairai et al., 2020; Filho et al., 2023). Currently, hydrogel microneedle technology demonstrates broad application prospects in fields such as local treatment of diabetic foot ulcers, vaccine delivery, chronic wound management, and biomarker monitoring (Yuan et al., 2026; Zhang et al., 2026; Yuan et al., 2022; Littauer et al., 2018; Zhang X. et al., 2024). The mechanism of action and drug release mechanism of hydrogel microneedles in Figure 4.

### 3.2 Structure of the microneedle hydrogel

**FIGURE 5:**
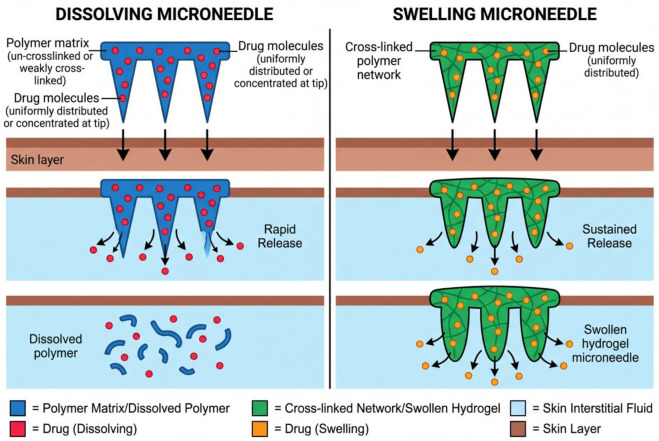
The mechanisms of action of different types of microneedles.

Hydrogel microneedles, as an important class of transdermal drug delivery systems, have structural design mechanisms that directly determine their behavior in the skin, thereby influencing drug release kinetics, biocompatibility, and clinical application outcomes. Based on the primary mode of interaction with skin interstitial fluid after insertion, hydrogel microneedles are generally classified into two types: dissolving and swelling (Figure 5).

#### 3.2.1 Structural design and mechanisms of dissolvable hydrogel microneedles

Dissolvable microneedle systems (DMNS) are generally fabricated from biocompatible materials such as polyvinylpyrrolidone (PVP) and hydroxypropyl methylcellulose (HPMC) (Shriky et al., 2023; Xuan et al., 2025). Their key advantage lies in their rapid dissolution upon insertion into the skin, facilitating efficient drug release (Lu et al., 2025; Xiang et al., 2025). When microneedles penetrate the skin, interstitial fluid quickly infiltrates the dry polymer network, resulting in hydration of the polymer chains. As hydration proceeds, the polymer molecules dissolve gradually, and drug release occurs via a dual mechanism involving diffusion and matrix erosion. The full process is generally completed within minutes, allowing for rapid drug delivery (Shriky et al., 2023). The release kinetics are affected by several factors, including the molecular weight of hyaluronic acid—studies indicate that under identical conditions, 74 kDa HA microneedles achieve an optimal balance between mechanical strength and transdermal release (Shriky et al., 2023). The release profile can usually be modeled using first-order kinetics.

#### 3.2.2 Structural design and mechanism of swellable hydrogel microneedles

Expandable hydrogel microneedles consist of cross-linked hydrophilic polymer networks. A key characteristic is their ability to swell significantly upon absorbing interstitial fluid, forming continuous hydrogel microchannels while the polymer network remains insoluble. After insertion into the skin, the dried microneedles hydrate by absorbing interstitial fluid, transforming into a soft hydrogel. Although the cross-linked structure prevents polymer dissolution, the swelling of the hydrogel increases the internal porosity of the matrix, thereby promoting the diffusion and release of drug molecules (Shriky et al., 2023; Mohite et al., 2024). Owing to the stability of the cross-linked network, drug release can be sustained over periods ranging from several days to weeks. MeHA microneedles are particularly suitable for extended-release applications due to their strong adhesive properties (approximately 0.20 N cm^-1^) and slow dissolution behavior (Shriky et al., 2023). The release mechanism generally follows non-Fickian diffusion, governed by both diffusion and matrix swelling, and the release rate can be precisely modulated by adjusting the cross-linking density—for instance, by varying the UV curing time (Shriky et al., 2023).

#### 3.2.3 Influence of microneedle design on drug delivery methods

In the structural optimization of microneedles, researchers enhance their performance in skin penetration and drug release efficiency by modifying geometric parameters and material composition. For instance, optimizing needle tip shape and shaft width can significantly reduce insertion force while improving drug delivery capacity (Lyu et al., 2023; Turner et al., 2023). Furthermore, microneedle design can be integrated with advanced technologies such as 3D printing, enabling the fabrication of personalized and complex architectures, thereby expanding their potential for clinical application (Li et al., 2022; Swain et al., 2023).

In summary, the distinctive structural and functional properties of microneedles endow them with considerable application potential in drug delivery, vaccination, and biological monitoring. Future research will continue to focus on optimizing material selection, enhancing structural design, and expanding clinical applicability. These efforts aim not only to improve drug delivery efficiency but also to further minimize patient discomfort, thereby facilitating broader clinical adoption.

### 3.3 Core advantages of the microneedle hydrogel

As a promising biomaterial, microneedle hydrogels have demonstrated significant potential in diabetic foot treatment owing to their unique physicochemical properties. Their high-water content, tunable mechanical and degradation characteristics, as well as stimuli-responsive release behavior—triggered by changes in temperature, pH, or enzyme activity—make them ideal for applications in advanced drug delivery systems and functional wound dressings (Xuan et al., 2025; Zhao et al., 2025; Peng et al., 2025).

Firstly, the high-water content inherent in hydrogels allows them to closely emulate the natural tissue microenvironment (Dong et al., 2025; Jiang et al., 2026). This moisture-rich nature not only confers excellent biocompatibility but also effectively maintains a moist wound environment, thereby facilitating the healing process (Liang et al., 2021; Shang et al., 2023). Studies have demonstrated that hydrogels exhibit outstanding wound healing efficacy in rat skin wound models, significantly accelerating tissue repair (Qiao et al., 2023).

Secondly, the mechanical and degradation properties of hydrogels can be precisely regulated by modulating their network structure and composition (Xiao et al., 2023; Liang et al., 2022). By introducing specific crosslinking agents or adjusting the molecular weight of the polymers, the strength and elasticity of hydrogels can be significantly enhanced (Guo et al., 2023). For instance, dual-crosslinked hydrogel systems can improve mechanical performance while maintaining biocompatibility, enabling them to remain stable under physiological loads (Noureen et al., 2023). Furthermore, the degradation rate of hydrogels can be controlled by varying the crosslinking density, which is critical for achieving sustained and controlled drug release in therapeutic applications (Guo et al., 2023).

**FIGURE 6:**
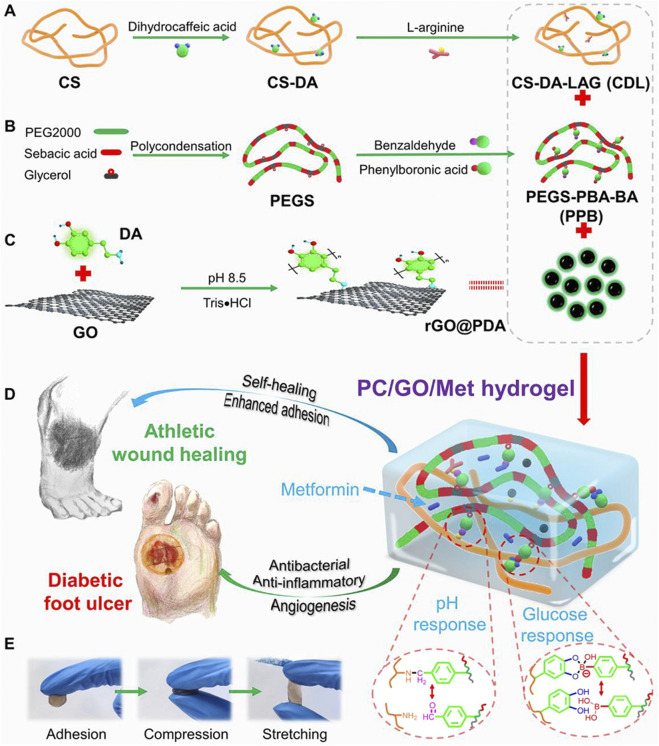
Schematic diagram of preparation and application of PC/GO/Met hydrogel. **(A)** Preparation of dihydrocaffeic acid and l-arginine cografting chitosan (CS-DA-LAG) and **(B)** phenylboronic acid and benzaldehyde difunctionalized polyethylene glycol-co-poly(glycerol sebacic acid) (PEGS-PBA-BA) and **(C)** polydopamine coated rGO (rGO@PDA). **(D)** The schematic diagram of structure, pH and glucose responsive mechanism of PC hydrogel and its application in diabetic foot ulcers and athletic wound healing. **(E)** Representative pictures of PC hydrogel for adhesion, compression, and stretching. Reprinted from Liang et al., (2022), with permission.

Finally, the stimuli-responsive drug release behavior of hydrogels represents a major advantage for their application in diabetic foot treatment. Certain hydrogels can react to environmental triggers such as temperature, pH, or specific enzymes, enabling intelligent and context-dependent drug release (Zheng and Xiao, 2023; Li Y. et al., 2024; Zhang G. et al., 2024). Liang et al. (2022) study, a pH/glucose dual responsive specific drug-metformin released multifunctional PEGS-PBA-BA/CS-DA-LAG (PC) hydrogel dressing was prepared via the double dynamic bonds of a Schiff base and dynamic phenylboronate ester between dihydrocaffeic acid and l-arginine cografted chitosan (CS-DA-LAG) and phenylboronic acid and benzaldehyde bifunctional polyethylene glycol-co-poly(glycerol sebacic acid) (PEGS-PBA-BA).This hydrogel exhibits excellent stability under neutral physiological conditions. When the glucose level and pH decrease in tissues, hydrolysis occurs, which alters the mechanical properties and simultaneously releases the encapsulated metformin to reduce the glucose level in the tissues (Liang et al., 2022) (Figure 6).The composite hydrogels rapidly release encapsulated drugs under high temperature or acidic conditions—a feature that not only enhances drug bioavailability but also minimizes systemic side effects (Lee, 2024). Moreover, smart responsive hydrogels can dynamically modulate drug release rates in accordance with changes in the wound microenvironment during healing, thereby supporting more precise and adaptive therapeutic interventions (Zhu et al., 2022; Liu et al., 2024b).

In summary, the distinctive properties of hydrogels establish them as highly suitable materials for the treatment of chronic wounds, including diabetic foot ulcers. Through continuous optimization of their composition and structure, the clinical potential of hydrogels is expected to expand further, particularly in the realms of personalized medicine and intelligent drug delivery systems.

## 4 Preparation and material selection of microneedles hydrogel

### 4.1 Common preparation methods

**FIGURE 7:**
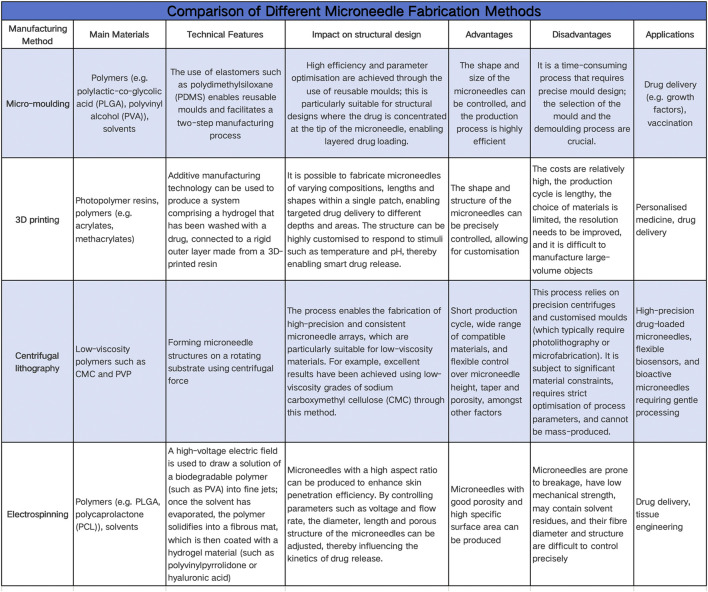
Shows a comparison of different microneedle preparation methods.

Microneedle hydrogels, an emerging class of materials for transdermal drug delivery and biomedical applications, can be prepared through a variety of methods. These generally involve critical steps including mold fabrication, formulation of the hydrogel precursor, cross-linking and curing, and post-processing stages such as demolding. The procedures are tailored to create microneedle arrays with defined geometries, mechanical robustness, and controlled drug release profiles, thereby enabling painless and efficient drug administration or diagnostic capabilities. Different manufacturing techniques directly influence the geometry, dimensional accuracy, material distribution, and functional integration capabilities of microneedles. The choice of manufacturing process not only determines the physical properties of microneedles but also affects their drug release kinetics, mechanical strength, and biocompatibility.Among these, micro-molding, 3D printing, and lithography (Davis et al., 2005) being the three most widely used techniques (Li et al., 2022; Economidou et al., 2018; Bagde et al., 2024; Razzaghi et al., 2024; Lee et al., 2008; Andranilla et al., 2023; Turner et al., 2021). Figure 7 shows a comparison of different microneedle preparation methods.

#### 4.1.1 Fabrication method based on micromolding

In the micro-molding approach, a hydrogel precursor solution is infused into a prefabricated mold and subsequently cured to form structured microneedle arrays. This method offers advantages such as simple equipment requirements, short production cycles, and suitability for large-scale manufacturing (Turner et al., 2021). However, a notable limitation is that repeated use of the mold may compromise product consistency and quality (Wu et al., 2023).

#### 4.1.2 Preparation methods based on 3D printing

3D printing technology enables the fabrication of more complex microneedle architectures, allowing precise control over geometry and size, as well as facilitating personalized design. By selecting appropriate biomaterials based on specific requirements, 3D-printed microneedle hydrogels can be tailored to achieve desired biocompatibility and mechanical properties (Ozyilmaz et al., 2021).

#### 4.1.3 Lithography-based fabrication methods

Lithography utilizes photochemical reactions in photosensitive materials to transfer predefined patterns into hydrogel structures, thereby forming microneedle arrays (Huang et al., 2024). This technique offers high precision and excellent reproducibility, yet it involves higher equipment costs and a relatively complex manufacturing process (Xie et al., 2022).

#### 4.1.4 Preparation methods for drug loading

In terms of drug loading strategies, microneedle hydrogels commonly employ physical blending, chemical conjugation, and nanocarrier encapsulation (Ding et al., 2024). Physical blending involves the direct mixture of drugs with hydrogel materials, making it suitable for straightforward loading of small-molecule drugs (Wang et al., 2018). However, this approach often lacks precise control over drug release kinetics, which may compromise therapeutic efficacy (Ping et al., 2023). Chemical conjugation covalently links drugs to the hydrogel matrix via chemical reactions, enhancing both drug stability and loading capacity (Xuan et al., 2025; Wang et al., 2018). This strategy is particularly suitable for long-term release applications, such as with bioactive molecules and proteins (Zhang Q. et al., 2024). Nanocarrier encapsulation entails encapsulating drugs within nanocarriers before incorporating them into the hydrogel system. This method improves drug bioavailability and targeting ability, thereby enhancing therapeutic outcomes (Chellathurai et al., 2021; Yao et al., 2024).

#### 4.1.5 Importance of sterilization methods and performance balance

Sterilization plays a critical role in preserving the material properties and drug activity of microneedle hydrogel systems (Zhong et al., 2026). During the preparation of microneedle systems (MNS), physical methods such as gamma irradiation or steam sterilization are commonly employed to ensure microbiological safety of the final product. The choice of sterilization method directly influences the physical integrity and drug release profile of the hydrogel. For instance, radiation sterilization may reduce the crosslinking density of hydrogels, potentially compromising their mechanical strength and modulating drug release kinetics (Su et al., 2024). Therefore, optimizing sterilization protocols to balance microbial safety and functional performance is essential in the fabrication of microneedle hydrogels.

In summary, the fabrication methods, drug loading strategies, and sterilization processes of microneedle hydrogels are critical factors influencing their clinical translation. Future research should prioritize the development of novel materials and the optimization of preparation protocols to enhance the therapeutic efficacy and patient compliance of microneedle hydrogel-based treatments for diabetic foot.

### 4.2 Development of material systems

The development of material systems is a critical factor in the application of microneedle hydrogels, particularly in the context of diabetic foot treatment. The selection of materials directly influences drug release behavior, wound healing efficacy, and patient comfort. Current studies indicate that both natural and synthetic polymers offer distinct biological advantages and design flexibility for such applications.

Firstly, natural polymers—such as hyaluronic acid, gelatin, and chitosan—exhibit significant advantages in terms of biocompatibility and biodegradability. Hyaluronic acid (HA) has gained popularity in diabetic wound healing owing to its exceptional moisture-retention capacity and ability to promote cellular migration. Studies have demonstrated that HA supports cell proliferation and migration, thereby accelerating the healing process (Wang et al., 2022). Gelatin is widely utilized in wound dressings due to its excellent biocompatibility and biodegradability, contributing effectively to tissue regeneration and reduced infection risk (Razif et al., 2025). As a natural polymer, chitosan possesses notable antibacterial properties and high biocompatibility, making it effective in inhibiting bacterial infections in diabetic foot wounds and facilitating healing (Chen X. et al., 2024).

In contrast, synthetic polymers such as polyethylene glycol (PEG) and poly(lactic-co-glycolic acid) (PLGA) provide greater design flexibility for microneedle hydrogels. PEG is widely used in the fabrication of microneedle hydrogels owing to its excellent biocompatibility, tunable mechanical strength, and high water solubility. Studies have shown that by modulating the crosslinking density and molecular weight of PEG, the drug release profile and mechanical properties of the resulting microneedles can be optimized, thereby enhancing their therapeutic efficacy in diabetic foot treatment (Lu et al., 2023). PLGA, renowned for its controllable biodegradability and adjustable release kinetics, is extensively employed in drug delivery systems. It enables precise regulation of drug release rates, contributing to improved treatment accuracy and effectiveness (Lyu et al., 2023).

Additionally, the use of composite materials has emerged as a new trend in microneedle hydrogel research. By integrating natural and synthetic polymers, synergistic effects can be achieved to optimize overall performance. For example,: studies have shown that combining hyaluronic acid with polyethylene glycol can enhance the mechanical strength and biodegradability of hydrogels while maintaining excellent biocompatibility (Wang et al., 2022). Such composite systems not only improve drug stability but also enhance drug penetration and release efficiency, thereby better addressing the therapeutic requirements of diabetic foot patients.

In conclusion, the advancement of material systems is critical to the application of microneedle hydrogels. By leveraging the complementary advantages of natural and synthetic materials, researchers can design more efficient and safer microneedle hydrogel-based therapies, opening new possibilities for diabetic foot treatment. Future research should continue to explore novel material combinations and modifications to further enhance the efficacy and clinical adaptability of microneedle hydrogels.

## 5 The mechanism of microneedle hydrogel in diabetic foot ulcer healing

### 5.1 Control blood sugar levels

**FIGURE 8:**
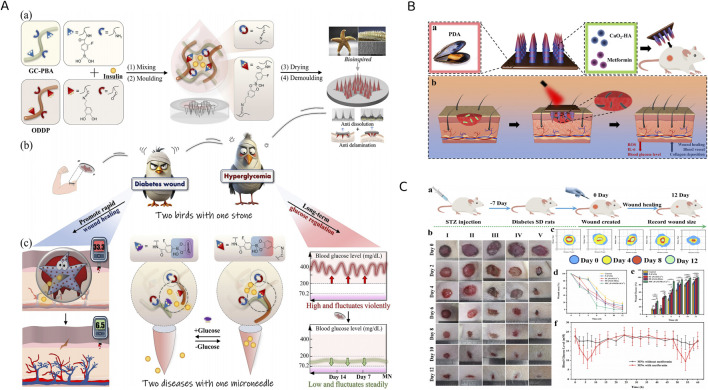
**(A)** Construction and application of an intelligent glucose-responsive microneedle system. Reprinted from Wang et al. (2026), with permission. **(B)** Schematic illustrations of MN patches for diabetic wound healing. Reprinted from Liu T. et al. (2023), with permission. **(C)** Effectiveness diagram of microneedle patches in promoting wound healing and controlling blood sugar. Reprinted from Liu T. et al. (2023), with permission.

The pathological process of diabetic foot ulcers involves multiple stages, with chronic hyperglycemia being the key etiological factor in the development of diabetic foot. Local hyperglycemia leads to an increase in advanced glycation end products, resulting in impaired vascular endothelial function, insufficient expression of inflammatory factors, compromised immune regulation, and reduced synthesis of extracellular matrix, all of which contribute to delayed wound healing. Controlling blood glucose levels is crucial in treating diabetic foot. Hydrogel microneedles can promote wound healing by locally administering drugs to regulate blood glucose levels. For example,: Yu et al. developed a dual-functional microneedle system with glucose responsiveness and the ability to mimic pancreatic insulin secretion (Wang et al., 2026). This system utilizes boronate ester bonds to achieve glucose-responsive release, while amine bonds enhance hydrogel stability and precisely regulate insulin release kinetics. Inspired by the morphology of starfish, the microneedle array was optimized to exhibit strong skin adhesion. This system demonstrates long-term blood glucose control and promotes wound healing (Figure 8A). Liu et al. developed a multilayer hydrogel microneedle patch loaded with calcium oxide and metformin (Liu T. et al., 2023). Inspired by porcupine quills, the patch features an optimized microneedle structure that not only provides strong tissue adhesion but also exhibits excellent antibacterial properties and blood glucose-lowering effects (Figure 8B). This multifunctional dressing, capable of reducing blood glucose and preventing infection, holds significant potential in promoting wound healing. *In vivo* experiments using a diabetic rat model demonstrated that compared to drug-free microneedles, the novel microneedle dressing significantly shortened wound healing time as blood glucose levels decreased (Figure 8C).

### 5.2 Regulation of the inflammatory microenvironment

**FIGURE 9:**
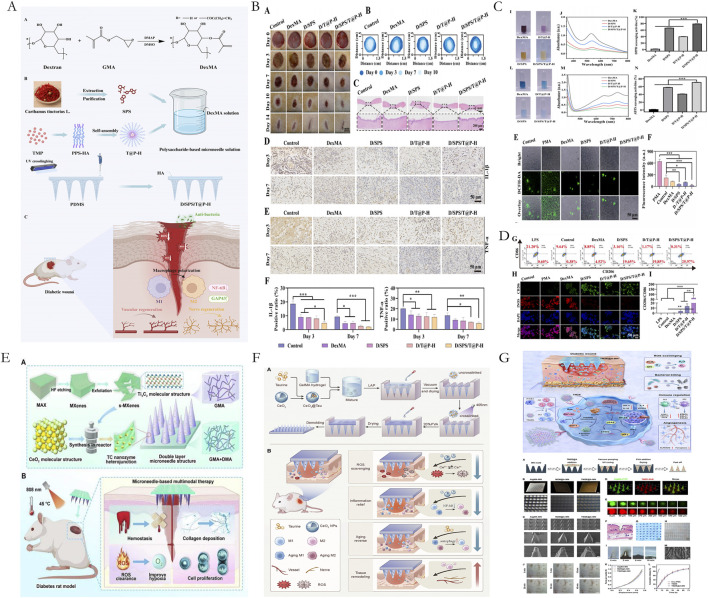
**(A)** Preparation process of D/SPS/T@P-H and its mechanism for promoting wound healing. Reprinted from Zhong et al. (2026), with permission. **(B)** Microneedles containing TMP and SPS have the effect of promoting wound healing. Reprinted from Zhong et al. (2026), with permission. **(C)** Hydrogels containing SPS and TMP can effectively eliminate ROS *in vitro* and *in vivo*, exerting antioxidant effects. Reprinted from Zhong et al. (2026), with permission. **(D)** Polarization of macrophage phenotypes. **(E)** The fabrication of bilayer microneedle patches incorporating Ti3C2/CeO2 (TC) nanozyme heterojunctions and DMA bio-adhesive molecules results in hemostatic and antioxidant functions, promoting wound repair through the clearance of reactive oxygen species (ROS) (Chen et al., 2026). **(F)** The preparation process of multifunctional CTH@MN patch by which the microneedle system exerts anti-inflammatory, antioxidant and wound healing promoting effects. Reprinted from Tian et al. (2024), with permission. **(G)** The preparation process of TMZE@A-MN and the degradation process and regulation of inflammatory cells after its application on the skin. Reprinted from Lu et al. (2026), with permission.

Chronic inflammation and oxidative stress in diabetic foot ulcers are important factors that hinder healing (Zhang Q. et al., 2025; Guo et al., 2024; Qi et al., 2024). Diabetic foot ulcers are typically characterized by prolonged inflammation, which can be alleviated by modulating inflammatory factors and macrophage polarization. Microneedle hydrogels regulate the inflammatory microenvironment through the delivery of Cytokines with anti-inflammatory effects and drugs, thereby promoting the wound healing process. Macrophages play a crucial role in wound healing, and their polarization state directly influences the formation of the inflammatory microenvironment. Studies have shown that microneedle hydrogels not only enable efficient transdermal drug delivery but also facilitate the polarization of macrophages toward the M2 phenotype. This shift promotes an anti-inflammatory response, accelerates tissue repair, and enhances wound healing (Song et al., 2025; Wong et al., 2025). For example,: Jin et al. developed a ROS-responsive hydrogel microneedle system for delivering tetramethylpyrazine (TMP) and safflower polysaccharide (SPS) to promote diabetic wound healing through antioxidant and anti-inflammatory effects (Zhong et al., 2026) (Figure 9A). The hydrophilic polysaccharide component of TMP not only has redox activity but also is ROS-responsive. This study demonstrated through *in vitro* and intracellular experiments that the hydrogel system based on SPS and T@P-H has significant antioxidant capacity. *In vitro* DPPH and ABTS experiments showed that the hydrogel containing SPS could effectively scavenge free radicals and cause the solution to decolorize. Intracellular ROS detection further confirmed that the microneedles containing SPS and T@P-H could significantly reduce intracellular ROS levels, and both exhibited a synergistic antioxidant effect, jointly protecting cells from oxidative damage (Figure 9C). Dextran/SPS/TMP@amphiphilic poly (propylene sulfide)-hyaluronic acid (D/SPS/T@P-H) could significantly inhibit the polarization of macrophages to the pro-inflammatory M1 phenotype (CD86^+^) and simultaneously promote their transformation to the anti-inflammatory M2 phenotype (CD206^+^) (Zhong et al., 2026). Flow cytometry and immunofluorescence results showed that the proportion of M1 in this treatment group was extremely low (0.31%), while the proportion of M2 was significantly increased (25.97%), and the CD206/CD86 ratio was the highest among all groups, proving that it could effectively regulate the immune microenvironment towards repair (Figure 9D). The determination of inflammatory factors in mice showed that the microneedle system could significantly reduce the expression of IL-1β and TNF-α (Figure 9B,F). D/SPS/T@P-H accelerates the repair of chronic wounds by regulating macrophage function and reducing local inflammation (Zhong et al., 2026) (Figure 9B).

In another study, Chen et al. developed an NIR-enhanced antioxidant nanozyme heterojunction hydrogel microneedle system (GTM) (Chen et al., 2026) (Figure 9E), which has dual functions of hemostasis and antioxidation. DMA (methacrylated dopamine) was utilized to achieve rapid wet tissue adhesion and physical wound closure; the loaded Ti3C2/CeO2 (TC) nanozyme heterojunction activated the CeO2 catalytic site (Ce4+ ↔ Ce3+ cycle) through the interfacial charge transfer of MXene and the local surface plasmon resonance (LSPR) effect under near-infrared irradiation (Chen et al., 2026), thereby significantly enhancing the ROS scavenging ability. The research results showed that the GTM system significantly promoted wound closure, alleviated excessive inflammatory responses, and enhanced tissue regeneration. In addition, Shen et al. devised a hydrogel microneedle system incorporating multifunctional nanoparticles (Tian et al., 2024) (Figure 9F). The microneedle patch can precisely and efficiently deliver CeO_2_@Tau to the deep layers of wound tissue (Tian et al., 2024). CTH@MN mitigates oxidative damage and inflammatory responses in macrophages by inhibiting the ROS/NF-κB signaling pathway. Simultaneously, CeO2@Tau@Hydrogel@Microneedle (CTH@MN) activates autophagy-mediated anti-aging activity, thereby establishing a favorable immune microenvironment for tissue repair. Both *in vitro* and *in vivo* tests confirmed that the CTH@MN patch can significantly promote diabetic wound healing (Tian et al., 2024) (Figure 9F). In recent research, Li et al. developed a dissolvable alginate methacryloyl-based microneedle patch functionalized with polypeptide CFLFLFK-NH2-coupled manganese/zinc ion metal-organic framework (MnZn-MOF) loading enalaprilat (Ena) (TMZE@A-MN) (Lu et al., 2026) (Figure 9G). Ena promotes neutrophil repolarization from pro-inflammatory N1 to anti-inflammatory N2 state by inhibiting nuclear factor (NF)-κB axis and activating Smad3 pathway (Lu et al., 2026). By taking advantage of the specific recognition of neutrophil membrane receptors by CFLFLFK-NH2, Ena can be targeted to inhibit the activation of neutrophils. In addition, MnZn-MOFs possess free radical-eliminating performance and can effectively combat the growth of methicillin-resistant *Staphylococcus aureus* and *Escherichia coli* (Lu et al., 2026). This microneedle system can exert anti-inflammatory, anti-infective, and wound repair-promoting effects during the early inflammatory stage of wound healing, facilitating the repair of diabetic wounds. In summary, the research indicates that the delivery of anti-inflammatory cytokines and drugs via hydrogel microneedles to regulate the inflammatory microenvironment holds great potential for promoting wound healing.

### 5.3 Promoting angiogenesis and tissue regeneration

**FIGURE 10:**
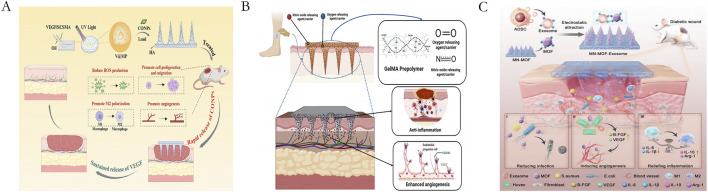
Preparation Process and Mechanism of Action Diagram of Microneedle Patches. **(A)** The V@MP/C@MN preparation process and its mechanism in promoting angiogenesis, scavenging reactive oxygen species, and regulating macrophage polarization for wound repair. Reprinted from He X. et al. (2024), with permission. **(B)** The development of NO and O2 releasing GelMA MN array from GelMA prepolymer and NO and O2 releasing donors. The Developed MN array patch enhanced wound healing by facilitating angiogenic and anti-inflammatory activity at the wound site. Reprinted from Ullah et al. (2025), with permission. **(C)** Schematic diagram of microneedle assembly and the mechanism of delivering MOFs and exosomes to the subcutaneous layer for their functional roles. Reprinted from Xiang et al. (2025), with permission.

The impaired healing of diabetic foot ulcers is also associated with impaired angiogenesis and insufficient tissue regeneration (Guo et al., 2024; Wang et al., 2022; He et al., 2025). Microneedle hydrogels can be loaded with pro-angiogenic factors, stem cells, or bioactive nanomaterials to ameliorate this situation (He et al., 2025; Zeng et al., 2017; Yan et al., 2023). Angiogenesis is a critical step in wound repair, and insufficient blood supply often leads to delayed healing. Research has demonstrated that microneedle hydrogels can significantly enhance neovascularization by releasing pro-angiogenic factors such as VEGF, thereby improving nutrient delivery to the wound site. For example,: Xinyue et al. developed a hydrogel microneedle system capable of dual-phase drug release (He X. et al., 2024). The encapsulated vascular endothelial growth factor hydrogel microspheres and cerium dioxide nanoparticles can promote wound healing by promoting angiogenesis and anti-inflammatory effects (Figure 10A). Cerium dioxide nanoparticles dissolve rapidly after insertion into the skin to reduce oxidative stress during the inflammatory stage and provide a favorable microenvironment for wound healing (He X. et al., 2024). Vascular endothelial growth factor can be released slowly to promote angiogenesis during the proliferative stage. The dual-phase drug release through hydrogel microneedles provides a new multifunctional synergistic treatment strategy for diabetic wound healing (He X. et al., 2024). Microneedles not only promote angiogenesis by releasing vascular endothelial factors but also facilitate the healing of diabetic wounds by encapsulating drugs and exosomes that enhance angiogenesis. For example,: Ullah et al. developed a visible-light-crosslinked hydrogel microneedle system for the delivery of NO and O_2_, which accelerates the healing of diabetic wounds by promoting angiogenesis and exerting anti-inflammatory effects (Ullah et al., 2025) (Figure 10B).Nitric oxide not only induces vasodilation by binding to soluble guanylate cyclase (sGC) (Ullah et al., 2025), but also modulates the mobilization of endothelial progenitor cells and promotes the maturation of newly formed vessels through the regulation of angiopoietin-1 and integrin signaling. Adequate O2 levels at the wound site are crucial for the function of immune cells, collagen synthesis, and tissue repair (Ullah et al., 2025). O2 can not only promotes the activity of macrophages and neutrophils, which help clear debris and prevent infection, but also can improve the hypoxic or low-oxygen levels at the wound site, control the production of ROS, and indirectly regulate the inflammatory response (Ullah et al., 2025). The dual-release GelMA MN array patch provides a promising therapeutic strategy for diabetic wound healing, enhancing angiogenesis, reducing inflammation, and accelerating tissue regeneration in a synergistic manner.

**FIGURE 11:**
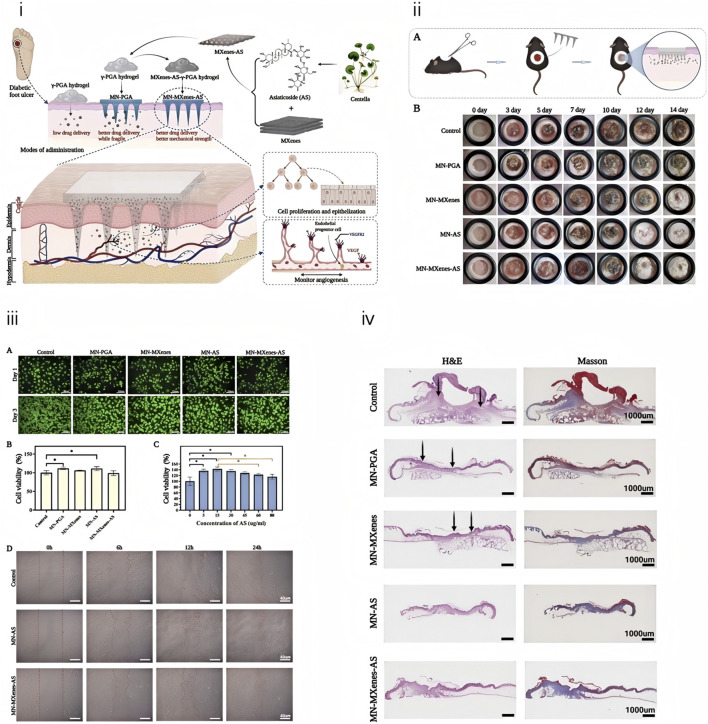
**(i)** Schematic illustration of MXenes-based microneedle patch (denoted as MN-MOF-GO-Ag) for accelerating diabetic wound healing. **(ii)** *In vivo* wound healing evaluation with diabetic mice. **(iii)** *In vitro* cell viability, migration and cytotoxicity of MN-MXenes-AS. **(iv)** Investigation on tissue regeneration and angiogenesis after treatments. Reprinted from (Wang et al. 2022), with permission.

In another study, Jieyu et al.developed a multifunctional MOF microneedle patch with adsorbed exosomes (Xiang et al., 2025). As the MNs degrade, the MOFs release both zinc ions, with antibacterial, anti-inflammatory, and angiogenic properties, and exosomes, which are internalized by cells and enhance cellular regeneration (Xiang et al., 2025) (Figure 10C). The RNA sequencing analysis shows that the patch accelerates wound healing by up-regulating key genes, activating ERK1/2 and PI3K-Akt signaling pathways, and promoting angiogenesis, cell migration and extracellular matrix remodeling (Xiang et al., 2025). In addition, Wang et al. study developed Ti2C3 MXenes integrated poly-γ-glutamic acid (γ-PGA) hydrogel microneedles to release asiaticoside to promote the healing of diabetic foot ulcers. Asiaticoside was encapsulated in PGMA hydrogel and enhanced its transdermal delivery ability through MXenes, which effectively promoted epithelization and angiogenesis (Wang et al., 2022) (Figure 11). *In vivo* experiments using a diabetic mouse model revealed that compared to the MN-PGA treatment group and the untreated group, wounds in diabetic mice treated with MN-AS and MN-MXenes-AS exhibited faster closure and more complete healing (Figure 11 ii, iv). *In vitro*, when human umbilical vein endothelial cells (HUVECs) and fibroblasts were co-cultured with extracts of MN-PGA, MN-AS, MN-MXenes, and MN-MXenes-AS, cell staining results demonstrated that MN-MXenes-AS significantly promoted the proliferation of both endothelial cells and fibroblasts (Figure 11iii). Together, these *in vivo* and *in vitro* findings indicate that MN-MXenes-AS possesses good biocompatibility and effectively accelerates the healing process of diabetic wounds.

Additionally, collagen remodeling constitutes another essential aspect of tissue regeneration. Microneedle hydrogels support collagen synthesis and reorganization by maintaining a conducive microenvironment (Guo et al., 2022; Hou et al., 2025). In several studies, these systems have been shown to accelerate the wound healing process and markedly increase collagen deposition, ultimately enhancing therapeutic outcomes.

In summary, the therapeutic benefits of microneedle hydrogels in diabetic foot wound healing rely on the precise spatiotemporal release of growth factors, targeted regulation of macrophage polarization, and the synergistic promotion of angiogenesis and collagen remodeling. These mechanisms not only provide novel strategies for clinical treatment but also establish a solid foundation for future basic research.

### 5.4 Anti-infection mechanisms

**FIGURE 12:**
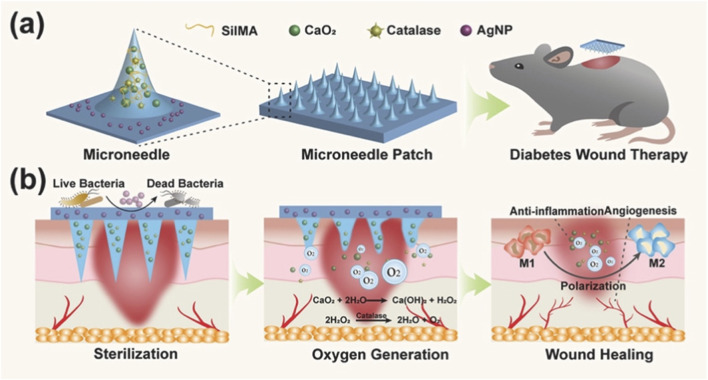
Antibacterial oxygen-generating microneedle (MN) patch for diabetic wound healing. **(a)** Schematic illustration of the MN patch. **(b)** Mechanism of the MN patch in wound healing. Reprinted from Sun et al. (2024), with permission.

Diabetic foot ulcers are often accompanied by bacterial infection, especially methicillin-resistant *Staphylococcus aureus* (MRSA) infection, which seriously hinders wound healing (Hu et al., 2024; Le et al., 2024). Microneedle hydrogels can be loaded with antimicrobial agents or antimicrobial nanoparticles to combat infection (Le et al., 2024; Liu et al., 2024c)]. For example,: an antibacterial oxygen-producing silk fibroin methacryloyl hydrogel microneed-needle patch based on calcium peroxide and catalase not only provides continuous oxygen supply, but also contains antibacterial silver nanoparticles, which effectively kill bacteria, inhibit biofilm formation, and reduce inflammation by regulating macrophage polarization (Sun et al., 2024) (Figure 12). In addition, the double-layer microneedle system has also been designed for immunomodulatory and infection response therapy to cope with persistent infection and immune dysfunction in diabetic foot ulcers (Chen et al., 2025) (Figure 12).

Infection control is critical in the management of diabetic foot, as infections not only impede wound healing but may also lead to severe complications such as amputation and even mortality. Consequently, the development of effective anti-infection strategies has become a major research focus. As an emerging drug delivery platform, microneedle hydrogels have demonstrated significant potential in combating infections (Chen et al., 2022), primarily manifested in the following aspects.

Firstly, localized high-concentration antibiotic delivery is recognized as an effective anti-infection strategy. Studies have shown that microneedle-based systems enable targeted antibiotic administration directly to the infection site, achieving high local drug concentrations. This approach not only reduces systemic side effects but also enhances drug efficacy within the infected area, thereby accelerating the healing process. One study demonstrated that antibiotics delivered via microneedles effectively inhibited the growth of *Staphylococcus aureus* and *Escherichia coli*, while promoting the healing of diabetic foot ulcers (Chen X. et al., 2024).

Secondly, the synergistic antibacterial effects of antimicrobial peptides (AMPs) and metal nanoparticles have been extensively investigated. AMPs are regarded as powerful agents against drug-resistant bacteria due to their unique mechanisms of action. When combined with metal nanoparticles, such as silver nanoparticles (AgNPs), they exhibit significantly enhanced anti-infective efficacy. For instance, one study demonstrated that a microneedle hydrogel incorporating both AMPs and AgNPs exhibited potent bactericidal activity against multiple drug-resistant bacterial strains *in vitro* and effectively promoted wound healing (Li Z. et al., 2024; Su et al., 2020). This strategy not only improves antibacterial performance but also reduces the risk of drug resistance development.

Targeting the penetration and disruption of bacterial biofilms represents a critical component of anti-infection strategies. Biofilms act as protective barriers formed by bacterial colonies during infections, conferring high resistance to both antibiotics and host immune responses. Therefore, developing microneedle hydrogels capable of effectively penetrating and disrupting biofilms is of particular importance. Studies have shown that dissolvable microneedle arrays functionalized with antimicrobial peptides can successfully deliver therapeutic agents into biofilm structures, enabling more comprehensive eradication of infections. Concurrently, this approach can stimulate the host immune response and accelerate wound healing (Su et al., 2020).

In conclusion, microneedle hydrogels demonstrate considerable promise in anti-infection strategies for diabetic foot wound management. By enabling localized high-concentration antibiotic delivery, leveraging the synergistic effects of antimicrobial peptides and metal nanoparticles, and targeting biofilm disruption mechanisms, this platform offers diverse and innovative approaches to control infections. These strategies not only enhance anti-infective efficacy but also open new avenues for future therapeutic development in diabetic foot care.

## 6 Optimization of drug delivery system for microneedle hydrogel

### 6.1 Single drug delivery system

In the treatment of diabetic foot, research on single-drug delivery systems has gained increasing attention, particularly regarding the application of small-molecule drugs, protein-based therapeutics, and nucleic acid agents. Firstly, the loading and release kinetics of small-molecule drugs such as metformin have been extensively studied. Evidence suggests that microneedle hydrogels can significantly enhance the transdermal delivery of metformin, resulting in improved drug release profiles. Studies have demonstrated that by incorporating specific polymers—such as polyvinyl alcohol (PVA) and polylactic acid (PLA)—into microneedle systems, sustained release of metformin can be achieved *in vivo*, thereby enhancing its therapeutic efficacy (Wang et al., 2022; Razif et al., 2025).

Next, strategies to stabilize protein-based drugs such as insulin require particular attention. Due to the susceptibility of protein therapeutics to degradation in external environments, hydrogel-based carriers can effectively preserve their bioactivity. Studies have indicated that incorporating insulin into specific hydrogel materials not only prolongs its release duration but also enhances its biological stability and efficacy. For example, research utilizing polyurethane-based hydrogels has successfully achieved sustained insulin release, significantly improving treatment outcomes in diabetic foot patients (Kumar et al., 2023; Barik et al., 2025).

In addition, enhancing the delivery efficiency of nucleic acid-based therapeutics, such as siRNA, represents a key research direction in single-drug delivery systems. Conventional siRNA delivery approaches are limited in clinical translation due to poor biocompatibility and low transfection efficiency. Studies have demonstrated that integrating nanoparticle carriers with microneedle platforms can significantly improve siRNA delivery performance. Nanoparticles not only enhance the stability of siRNA but also optimize its release profile within target tissues (Serrano-Castañeda et al., 2023; Alavi et al., 2024).

In summary, single-drug delivery systems demonstrate promising application prospects in the treatment of diabetic foot. By optimizing drug loading and release kinetics, implementing effective stabilization strategies, and enhancing the delivery efficiency of nucleic acid therapeutics, these systems can provide more effective treatment options for diabetic foot patients. Future research should continue to explore novel materials and technologies to better address the clinical demands of diabetic foot management.

### 6.2 Combined drug delivery system design

In the treatment of diabetic foot, the design of combined drug delivery systems is an important research direction, which aims to improve the sequential release and interaction of drugs to enhance efficacy and reduce side effects. In view of the special pathological environment of diabetic foot, researchers have proposed a variety of combination drug delivery strategies to improve the therapeutic effect.

Firstly, the sequential release of anti-inflammatory drugs and pro-healing factors is the core of the design of the combined drug delivery system. Recent studies have shown that the healing process of diabetic foot is often accompanied by chronic inflammation. Anti-inflammatory drugs such as Fluocinolone can effectively suppress the inflammatory response, while pro-healing factors such as Pyrrolidine can promote cell proliferation and angiogenesis. By regulating the release timing of these two drugs, the optimal balance between inflammation control and tissue regeneration can be achieved. For example, certain novel hydrogel systems are able to achieve the release of different drugs based on external stimuli, such as pH changes, thus providing anti-inflammatory drugs during the most severe phase of inflammation, while after inflammation is reduced, pro-healing factors are instead released (Wang et al., 2022).

Secondly, antibiotics and biofilm dispersant synergy formula is also an important part of combined medication. Infection is a common and serious complications in patients with diabetic foot, antibiotics such as Cephalosporin (Cephalosporin) can effectively control the infection, and biofilm dispersants such as poly (vinyl alcohol) (PVA) helps to enhance the permeability of antibiotics and bioavailability. Studies have found that complexes combining antibiotics and biofilm dispersants can significantly improve the inhibitory effect on multidrug-resistant bacteria *in vitro* models, and have shown good safety and efficacy in clinical trials (Razif et al., 2025).

Finally, the compatibility of multi-drug loading and the regulation of release curve are another important aspect in the design of combined drug delivery system. The compatibility of different drugs directly affects the effect of their combined application. By optimizing the drug loading ratio and release curve, the drug effect can be maximized. Certain studies have used nanocarrier technology in which multiple drugs are simultaneously loaded on the same carrier to achieve synergistic effects. This method not only improves the targeting of drugs, but also can control the release rate of drugs by adjusting the physicochemical properties of the carriers (such as particle size, surface charge, etc.), so as to provide the required drugs in different treatment stages (Jiang et al., 2023).

In conclusion, the design of combined drug delivery systems has shown great potential in the treatment of diabetic foot. Through the rational collocation and sequential release optimization of anti-inflammatory drugs, pro-healing factors, antibiotics, and biofilm dispersants, not only the therapeutic effect can be improved, but also the side effects can be reduced, and more effective treatment options can be provided for patients. In the future, with the continuous development of materials science and drug delivery technology, combined drug delivery systems are expected to achieve more significant results in clinical application.

## 7 Challenges and strategies in the application of microneedle hydrogel in diabetic foot ulcer healing

### 7.1 Challenges in clinical translation

#### 7.1.1 Technical bottlenecks

In the application of microneedle hydrogels, particularly for diabetic foot treatment, addressing technical bottlenecks is critically important. A primary challenge lies in quality control during large-scale production. The fabrication process involves complex material formulations and precise manufacturing steps, where even minor deviations can compromise the safety and efficacy of the final product. For instance, studies have indicated that drug release profiles and cytocompatibility of microneedle hydrogels are highly sensitive to variations in material composition and structural uniformity (Wang et al., 2022). Therefore, establishing standardized quality control protocols to ensure batch-to-batch consistency and product stability is essential for advancing this technology toward clinical translation.

Secondly, the impact of sterilization processes on material properties cannot be overlooked. When used in diabetic foot treatment, microneedle hydrogels must undergo rigorous sterilization to eliminate potential microbial contamination. However, sterilization may adversely affect the physicochemical properties of the hydrogel, potentially compromising its biocompatibility and *in vivo* drug release performance (Richter et al., 2023). Therefore, developing novel sterilization methods or optimizing existing protocols to minimize detrimental effects on hydrogel functionality is crucial for overcoming this bottleneck.

Finally, the balance between storage stability and ease of use is also an important technical bottleneck. The storage conditions of microneedle hydrogel directly affect its validity and application effect. Studies have pointed out that the moisture content and physical state of hydrogels can change during storage, which may lead to a decrease in their drug release performance (Long et al., 2022). To this end, developing materials with better storage stability or optimizing packaging design for easier use in clinical conditions are potential directions to enhance microneedle hydrogel technology.

In summary, the application of microneedles hydrogels in the treatment of diabetic foot faces several technical bottlenecks, including production quality control, the effect of sterilization process on material properties, and the trade-off between storage stability and ease of use. Future research needs to focus on solving these bottlenecks to promote the clinical translation and application of microneedle hydrogel technology.

#### 7.1.2 Barriers to clinical application

The clinical application of microneedle hydrogels in the treatment of diabetic foot (DFU) faces multiple obstacles, including the difficulty in achieving individualized treatment, the heterogeneity of chronic wounds, and the issues of medical cost and medical insurance coverage.

First of all, the difficulty of implementing individualized treatment is a significant barrier. Patients with diabetic foot often have multiple complications and individual differences, such as glycemic control, blood circulation status, and immune response, which complicate the design of treatment programs (Alsarara et al., 2025). In addition, the healing process of chronic wounds varies depending on the patient’s overall health status, lifestyle habits, and comorbidities, making it challenging to develop an individualized treatment plan that is suitable for each patient. For example, some patients may respond well to a particular treatment, while others may not achieve the same results due to their unique pathophysiological features (Mohsin et al., 2024). This heterogeneity leads to unpredictable therapeutic effects, which makes clinicians need to be cautious when choosing microneedle hydrogel as a treatment method.

Secondly, the effect of heterogeneity of chronic wounds on the efficacy cannot be ignored. Chronic wounds in diabetic foot are usually accompanied by different pathophysiological states, such as infection, ischemia, and neuropathy, which all affect the speed and quality of healing (Long et al., 2022; Benadda et al., 2025). Micro needle hydrogel although brought the good curative effect in the laboratory, but in a complex clinical environment, due to the complexity of the diversity of the wound and the healing mechanism, therapeutic effect may be very different. In addition, the risk of bacterial infection should be considered in the treatment of chronic wounds, and the application of microneedles hydrogel needs to be carried out under sterile conditions to avoid the occurrence of secondary infection (Jiang et al., 2023).

Finally, healthcare costs and health coverage problem is also important obstacle of the popularization and application of the micro needle hydrogel. Although microneedle hydrogel has potential economic benefits in the treatment of diabetic foot, its high initial development and production costs may lead to a large financial burden for patients when receiving treatment (Gopal et al., 2025). In addition, the coverage of medical insurance policies is limited, and many patients may not be able to afford the cost of out-of-pocket treatment. Under the influence of these factors, the clinical application of microneedles hydrogel is limited. Therefore, how to reduce manufacturing costs and improve medical insurance policies to promote the application of microneedle hydrogel in the treatment of diabetic foot has become an important direction for future research and policy formulation (Wu et al., 2023).

To sum up, the micro needle hydrogels in clinical application in the treatment of diabetic foot obstacle lies in the complexity of individualized treatment, the heterogeneity of chronic wounds and medical costs and health coverage. To solve these obstacles to collaborative, including strengthening basic research, to optimize the design of the clinical trials, reduce the cost and promote the policy reform.

#### 7.1.3 Regulatory considerations

As a novel drug delivery system, HMNs face a complex regulatory pathway, requiring compliance with both medical device and drug (or biological product) regulations. Before microneedle technology can be widely adopted, relevant regulatory guidelines are still under development.

Material safety and biocompatibility: Hollow microneedles (HMNs) are commonly fabricated from natural or synthetic polymers (Shriky et al., 2023). Comprehensive evaluation—including assessment of degradation profiles, biocompatibility, and potential local or systemic toxicity—is essential for both *in vitro* and *in vivo* toxicological characterization.1, For example,: gelatin methacryloyl (GelMA) hydrogel-based microneedles have enabled effective transdermal delivery of metformin in a diabetic rat model, resulting in significant modulation of blood glucose levels (Zeng et al., 2021).Nevertheless, clinical translation of any novel HMN material necessitates robust, human-relevant safety data to substantiate long-term biocompatibility and absence of cumulative toxicity (Zeng et al., 2021).

Sterility assurance: As invasive medical devices, HMNs must maintain sterility during manufacturing, packaging, storage, and use.8 The selection of appropriate sterilization methods is critical, as these methods must effectively eliminate microorganisms without compromising the structural integrity, drug activity, or mechanical properties of the hydrogel (Shriky et al., 2023). For instance, certain sterilization techniques (e.g., gamma irradiation) may damage the crosslinked structure of the hydrogel (Shriky et al., 2023).

Mechanical properties and delivery efficiency: Microneedles must possess sufficient mechanical strength to reliably penetrate the stratum corneum, achieve precise drug deposition in the viable epidermis or dermis, and avoid structural failure (e.g., buckling or fracture) or unintended tissue damage during insertion.6 8 To address this challenge, recent strategies focus on nanomaterial reinforcement—such as incorporation of cellulose nanocrystals or silica nanoparticles—and rational geometric design to improve the mechanical robustness of hollow microneedles (HMNs), thereby reconciling high insertion force tolerance with controlled post-insertion swelling behavior (Golshirazi et al., 2024).6.

Product quality and stability: Adoption of the Quality by Design (QbD) framework is essential for regulatory approval of advanced microneedle-based delivery systems, enabling systematic identification of critical quality attributes (CQAs) and robust control of manufacturing variability to ensure batch-to-batch consistency and clinical performance.8 Furthermore, the long-term storage stability of hollow microneedles (HMNs)—particularly the effects of cyclic dehydration and rehydration on needle geometry, mechanical integrity, and drug payload retention—remains a key knowledge gap requiring rigorous experimental characterization to guarantee product quality, potency, and safety throughout the labeled shelf life (Shriky et al., 2023).

Evaluation of combination products: When hollow microneedles (HMNs) incorporate drugs or bioactive agents, regulatory-grade *in vitro* and *in vivo* pharmacokinetic (PK) and pharmacodynamic (PD) studies are mandatory to demonstrate not only efficient transdermal drug delivery but also spatiotemporally controlled release and functional responsiveness within the dynamic wound microenvironment (Wang et al., 2023; Guo et al., 2022). Comprehensive PK/PD datasets—including absorption kinetics, tissue distribution profiles, target engagement biomarkers, and functional outcomes—are essential for regulatory evaluation of safety, efficacy, and clinical relevance (Wang et al., 2023; Guo et al., 2022).For example,: glucose-responsive hydrogel microneedle dressings have been engineered to autonomously modulate insulin release and promote accelerated re-epithelialization in a type 1 diabetic murine wound model (Guo et al., 2022).

#### 7.1.4 Scalability

Scalable manufacturing of hollow microneedles (HMNs) remains a critical bottleneck for clinical translation. Conventional microfabrication methods—including soft lithography, replica molding, and template-based casting—are often labor-intensive, low-throughput, and constrained by resolution limits (<50 µm), thereby impeding high-yield, cost-effective production of structurally uniform HMNs suitable for regulatory-grade clinical use (Zhou et al., 2024a; Zhou et al., 2024b).

Manufacturing process innovation: Digital Light Processing (DLP) 3D printing represents a paradigm shift in hollow microneedle (HMN) fabrication, enabling rapid, high-precision manufacturing of temperature-responsive hydrogel microneedles with feature resolution below 50 µm. This technique supports on-demand customization of geometric parameters—including needle height, base diameter, taper angle, and array configuration—within approximately 1 h per batch, while preserving structural fidelity across diverse designs (Zhou et al., 2024a).Crucially, DLP leverages nanocomposite hydrogel precursor inks—formulated to balance photoreactivity, mechanical integrity, and biocompatibility—thereby circumventing the mold dependency, resolution constraints, and scalability limitations inherent to conventional microfabrication methods, and offering a viable route toward GMP-aligned production (Zhou et al., 2024b).

Inter-batch consistency: Even with advanced manufacturing techniques, ensuring that each batch of produced HMNs maintains high consistency in terms of size, shape, mechanical strength, drug loading, and release characteristics is still the foundation for meeting regulatory requirements and ensuring clinical efficacy.

Long-term storage stability: The long-term storage stability of HMNs—particularly the preservation of needle morphology, mechanical functionality, and drug payload integrity following dehydration–rehydration cycling—requires systematic investigation under internationally harmonized conditions (e.g., ICH Q5C). Robust stability data are necessary to define an evidence-based shelf life, confirm chemical and physical stability of the active ingredient, and validate reliable reconstitution behavior prior to clinical use (Shriky et al., 2023).

#### 7.1.5 Cost-effectiveness

The treatment of diabetic foot ulcers (DFUs) is itself a significant economic burden. Although hydrogel matrix nanocomposites (HMNs) as a novel therapy may have higher initial production costs than traditional dressings, their potential clinical advantages may lead to long-term cost savings.

Potential long-term benefits: If HMNs can effectively shorten the healing period of DFUs, reduce amputation rates and readmission rates, then despite the higher initial costs, their overall health economics benefits may be superior. For instance, amputation not only causes great suffering to patients but also incurs high medical expenses. By accelerating healing, HMNs are expected to reduce these indirect costs.

Lack of economic evaluation: Currently, there is a relative lack of high-quality health economics analyses on the cost-effectiveness of HMNs in the treatment of DFUs. Future research needs to conduct real-world studies to verify the potential gains of HMNs in improving patients' quality-adjusted life years (QALYs) and comprehensively assess their impact on the total cost of the healthcare system. The cost-effectiveness evaluation of ON101 cream (a new treatment method for promoting DFU wound healing) shows that it may be cost-effective compared to conventional wound care alone, providing a reference framework for evaluating the cost-effectiveness of HMNs.

#### 7.1.6 Patient compliance

Patient compliance is crucial for the effective treatment of DFUs (Pouwer et al., 2023; Ng et al., 2024). As a minimally invasive and painless drug delivery method, HMNs are expected to enhance patient acceptance and compliance (Rathee and Dayaramani, 2025; Smith et al., 2022).

Ease of operation: Compared with traditional methods such as multiple daily insulin injections, the micro-needle technology offers greater convenience and comfort, which is expected to improve patient acceptance and compliance (Smith et al., 2022). However, although HMNs aim to simplify the operation, correct application and removal may still require professional guidance, especially for elderly or mobility-impaired DFU patients (Ng et al., 2024).

Replacement frequency: The replacement frequency of HMNs (typically 24–72 h) may require regular operation by patients or caregivers, which may have an impact on daily life (Shriky et al., 2023).Optimizing drug release kinetics and extending the *in-situ* time of micro-needles can further improve patient compliance.

Dependency on wound condition: Severe DFUs are often accompanied by edema, necrotic tissue or biofilm formation, which may hinder the effective penetration of micro-needles and affect drug delivery efficiency (Mo et al., 2023). The formation of biofilms can seriously affect wound healing, and the combination of HMNs with nanomaterials can synergistically address this issue and enhance transdermal treatment effects (Golshirazi et al., 2024; Chen Z. et al., 2024).

Patient education and self-management: The self-care ability of diabetic patients is crucial for the prevention and treatment compliance of DFUs (Pouwer et al., 2023; Mohammed, 2024).Elderly DFU patients may have insufficient self-management ability due to cognitive impairment or cultural differences, and need to be strengthened in education and support (Mohammed, 2024). For example,: foot decompression is a key in the treatment of DFUs, but patient compliance is often low. This situation can be improved through technological progress and patient education (Bus et al., 2024; Racaru et al., 2022; Zhu et al., 2025).

#### 7.1.7 Technological innovation path

With the continuous development of the field of diabetic foot treatment, the innovation path of microneedle hydrogel technology has become particularly important. These innovations not only improve the treatment effect, but also provide patients with more convenient treatment options.

Firstly, the development of responsive smart microneedle hydrogels brings new hope for the treatment of diabetic foot. Such microneedle hydrogels can intelligently release drugs in response to changes in the environment, such as pH, temperature, or concentration of biomarkers. For example, studies have shown that MXenes-integrated microneedle hydrogels are able to effectively release drugs and promote cell proliferation and angiogenesis, which are critical for improving the healing process of diabetic foot ulcers (Wang et al., 2022). The design of this microneedle not only improves the transdermal absorption rate of the drug, but also its enhanced mechanical strength allows it to effectively penetrate the cuticle, ensuring the timely release of the drug, thereby accelerating the healing of diabetic foot ulcers.

Secondly, combining with the emergence of sensing technology integration system of diagnosis and treatment, make the management of diabetic foot more intelligent. This system through the integrated sensor and the micro needle technology, can real-time monitor the patient’s health status, and automatically adjust according to the results of the test drug release. This innovative approach not only improves the level of personalization of treatment, but also enhances patient compliance. For example, using micro needle sensor can continuously monitor blood glucose level in diabetic patients, adjust the treatment plan, and according to the real-time data to optimize the management of diabetic foot (Razif et al., 2025).

Finally, the application of 3 days printing personalization solutions for the development of micro needle hydrogel opened up a new direction. 3D printing technology can quickly produce microneedle hydrogels that meet individual needs according to the specific conditions of patients. Such customized microneedles can be optimized for different types of diabetic foot ulcers, thereby improving the efficacy and safety of treatment. For example, researchers have successfully used 3D printing technology to develop microneedle hydrogels with excellent mechanical properties and biocompatibility, which can effectively promote wound healing after application and have good antibacterial properties (Atoum et al., 2023). This personalized treatment approach will undoubtedly provide a better nursing experience for patients with diabetic foot.

In summary, the technological innovation path of microneedle hydrogel in the treatment of diabetic foot covers many aspects, such as intelligent response, combination of sensing technology and personalization. These innovations not only promote the treatment process of diabetic foot, but also lay the foundation for achieving higher patient satisfaction and quality of life. With the deepening of related research and the continuous improvement of technology, microneedles hydrogel will play a more important role in the clinical application of diabetic foot in the future.

### 7.2 Strategies for clinical translation

In the micro needle hydrogel clinical transformation, indications and precise medical segment is the base of implement effective treatment. Micro needle hydrogel as a new drug delivery system, has good biocompatibility and controlled drug release properties. This makes it show great potential in the local treatment of chronic diseases such as diabetic foot. As the research on microneedle hydrogels continues to deepen, the collection and analysis of clinical trial data become crucial. In this study, for a specific population (such as diabetes) hierarchical analysis can help identify different individual differences in response to treatment, so as to realize precise medical care. For example, studies have shown that microneedle hydrogel can effectively improve the healing effect of patients with diabetic foot, and this effect may be closely related to the specific pathological state, age and comorbidities of patients (Sharma M. B. et al., 2024). By integrating the microneedle hydrogel with the clinical characteristics of individuals, doctors can develop more personalized treatment plans and improve the success rate of treatment.

The collection and analysis of real-world data is another key strategy for clinical translation of microneedle hydrogels. Real-world research (RWE) can provide a wider range of clinical application data to help evaluate the efficacy and safety of microneedle hydrogels in practical applications. These data will not only reflect the performance of microneedle hydrogels outside laboratory conditions, but also reveal their applicability in different populations. For example, by long-term follow-up of diabetic patients using microneedle hydrogel, its efficacy and side effects in different environments and lifestyles can be evaluated (Turner et al., 2023). Such data collection will provide an important basis for market access and clinical guideline development of microneedling hydrogels.

The collaborative innovation model of industry, university, research and medicine is an effective way to promote the clinical translation of microneedle hydrogel. The cooperation between scientific research institutions and medical institutions can not only accelerate the clinical application of new technologies, but also solve clinical needs by jointly developing products and technology platforms. Based on the latest scientific research results, enterprises can develop microneedle hydrogel products with higher safety and effectiveness, thereby promoting their application in diseases such as diabetic foot (He Y. et al., 2024). In addition, the feedback from medical institutions and clinical data can in turn promote the deepening of scientific research, forming a virtuous cycle to promote the continuous progress of microneedle hydrogel technology and the expansion of clinical applications.

In summary, the clinical translation strategy of microneedle hydrogel requires a combination of indication segmentation and precision medicine, the collection and analysis of real-world data, and the collaborative innovation model of industry, university, research and medicine. Through the implementation of these strategies, the application of microneedle hydrogel in the treatment of diabetic foot can be better promoted, and finally the quality of life and treatment effect of patients can be improved.

## 8 Future development direction of microneedle hydrogel in diabetic foot ulcer healing

### 8.1 Research and development of new materials

#### 8.1.1 Intelligent responsive materials

To develop microneedle hydrogel materials with multiple intelligent response properties (such as pH response, temperature response, enzyme response, etc.) to enable more precise drug release and treatment effect in the specific microenvironment of diabetic foot ulcers. For example, taking advantage of the reduced pH value in the local microenvironment of diabetic foot ulcers, pH-responsive microneedle hydrogels are designed to specifically release drugs at the ulcer site and improve the targeting of treatment.

#### 8.1.2 Bioactive materials

To find and develop materials with higher biological activity, such as those containing natural bioactive ingredients (such as peptides and polysaccharides), which can not only serve as drug carriers, but also participate in the tissue repair process by themselves and play a variety of biological functions to further improve the effect of micro-needle hydrogels in promoting ulcer healing.

### 8.2 Optimization of preparation process

#### 8.2.1 Improvement of microneedle preparation technology

Explore new microneedle preparation technology or improve the existing technology to improve the accuracy and efficiency of microneedle preparation. For example, the use of 3D printing technology can realize the personalized customization of microneedles, and microneedle hydrogels with specific size, shape, and drug loading can be prepared according to the specific conditions of patients.

#### 8.2.2 Innovation of drug loading and release technologies

New drug loading and release technologies are developed, such as the use of nanotechnology to encapsulate drugs in nanoparticles and then load them on microneedle hydrogels. The sustained release characteristics of nanoparticles can further prolong the release time of drugs and improve the therapeutic effect of drugs. At the same time, a technology that can simultaneously load multiple drugs and achieve sequential release is developed to meet the therapeutic needs at different stages of diabetic foot ulcer healing.

### 8.3 In-depth clinical research

#### 8.3.1 Multi-center and large-sample clinical trials

Large-scale and multi-center clinical trials will be carried out to further verify the efficacy and safety of microneedle hydrogel in the treatment of diabetic foot ulcers. Through rigorous clinical trial design and data analysis, the best use plan of microneedle-based hydrogel is clarified, including treatment cycle, drug dose, and frequency of use, so as to provide a more reliable basis for clinical application.

#### 8.3.2 Combined treatment strategy

Explore the combined application of microneedle hydrogel with other treatment methods (such as debridement, antibiotic therapy, hyperbaric oxygen therapy, etc.) to play a synergistic role of different treatment methods and improve the therapeutic effect of diabetic foot ulcer. For example, the use of microneedle hydrogel loaded with antibiotics for local treatment after debridement can not only effectively control infection, but also promote ulcer healing.

## 9 Conclusion

As a new type of biological material, microneedle hydrogel has shown great potential in the field of diabetic foot ulcer healing. Its unique structure and properties enable it to achieve efficient drug delivery, regulate cell behavior and inflammatory response, thereby promoting ulcer healing. Although there are still some challenges in materials, preparation process, and clinical translation, with the development of new materials, optimization of preparation process, and in-depth clinical research, microneedle-based hydrogel is expected to become an effective means for the treatment of diabetic foot ulcers, bringing new hope to the vast number of diabetic patients. In the future, multidisciplinary cooperation is needed to jointly promote the development of microneedle hydrogel in the field of diabetic foot ulcer treatment, and make it move from laboratory to clinical application as soon as possible.
